# Sodium Alendronate-Modified PLGA-mPEG Nanomicelles Loaded with Rifapentine for Targeted Delivery to Bone Tissue

**DOI:** 10.3390/pharmaceutics18030352

**Published:** 2026-03-12

**Authors:** Weilin Wang, Xin Cui, Hengfa Wei, Jingjing Wang, Yesbolat Ahehati, Cuiping Jiang, Fei Li, Shasha Li

**Affiliations:** 1 State Key Laboratory of Pathogenesis, Prevention and Treatment of High Incidence Diseases in Central Asia, College of Pharmacy, Xinjiang Medical University, Urumqi 830054, China; wangweilinww@163.com (W.W.);; 2 Guangdong Provincial Key Laboratory of Chinese Medicine Pharmaceutics, School of Traditional Chinese Medicine, Southern Medical University, Guangzhou 510515, China; 3 State Key Laboratory of Natural Medicines, China Pharmaceutical University, Nanjing 210009, China; 4 Xinjiang Key Laboratory of Natural Medicines Active Components and Drug Release Technology, Urumqi 830054, China

**Keywords:** bone targeting, drug delivery system, polymeric micelles, bisphosphonates, rifapentine

## Abstract

**Background/Objectives:** The limited targeting efficiency and systemic toxicity of conventional medicine present significant challenges in the treatment of skeletal disorders, such as bone tuberculosis. To address these limitations, we developed a bone-targeting nanomicelle delivery system functionalized with alendronate (ALN), designated ALN-PLGA-mPEG@RPT, to improve the targeted delivery and therapeutic efficacy of rifapentine (RPT) in bone tissue. **Methods:** The ALN-PLGA-mPEG blank micelles, prepared in accordance with our research group’s optimized protocol, were loaded with RPT and subjected to systematic formulation optimization. The resulting nanomicellar system was comprehensively characterized in terms of its physicochemical properties, including particle size and polydispersity index (PDI). Additionally, drug-loading capacity, encapsulation efficiency, and in vitro release curve were evaluated. Bone-targeting efficacy was assessed using in vivo imaging techniques, while biodistribution and safety profiles were determined through in vivo distribution studies and histopathological examination. **Results:** The optimized ALN-PLGA-mPEG@RPT nanomicelles exhibited a mean particle size of 101.90 ± 4.17 nm, and a PDI of 0.242 ± 0.021. The formulation achieved a drug loading of 16.74 ± 0.51% with an encapsulation efficiency of 50.27 ± 1.91%. In vitro release studies confirmed a sustained-release profile, with only 25% of RPT released within 12 h. In vivo imaging revealed significantly enhanced bone-targeting capability in the ALN-modified group, showing a 1.93-fold higher drug accumulation in bone tissue compared to blood. Histopathological analysis indicated no observable pathological alterations in major organs. **Conclusions:** The ALN-PLGA-mPEG@RPT nanomicelle system exhibits favorable bone-targeting efficiency, sustained-release properties, and biocompatibility, representing a promising strategy for the precise treatment of bone tuberculosis and other skeletal diseases.

## 1. Introduction

Tuberculosis (TB), a chronic infectious disease caused by Mycobacterium tuberculosis, remains a serious threat to global public health security. According to the World Health Organization’s (WHO) Global Tuberculosis Report 2025, approximately 10.7 million people worldwide were affected by TB. TB remains one of the top ten causes of death globally and is the leading cause of death from a single infectious agent, claiming nearly twice as many lives as HIV/AIDS—a situation that remains grave [1,2]. It is estimated that about one-third of the global population harbors latent M. tuberculosis infection. This pathogen can remain dormant within the protective microenvironment of the host immune system for prolonged periods, posing a substantial latent risk [1,3].

Among the various extrapulmonary manifestations of TB, osteoarticular tuberculosis stands as one of the most prevalent forms. Spinal tuberculosis, which represents the most common subtype of skeletal TB, can lead to irreversible neurological deficits, kyphotic deformity, and lifelong disability, imposing a substantial burden on both affected individuals and society [4]. Current treatment for bone tuberculosis primarily involves surgical intervention and pharmacological therapy. However, even following successful surgery, patients are generally required to adhere to at least one year of oral anti-tuberculosis chemotherapy [5,6]. Nevertheless, due to the inherently poor vascularization of bone tissue and the frequent presence of necrotic and calcified lesions, conventional oral medications often fail to achieve therapeutic concentrations at the infection site [7,8]. Moreover, prolonged multidrug regimens involving high doses are prone to cause severe adverse effects such as hepatotoxicity and gastrointestinal disturbances, leading to suboptimal treatment adherence and high rates of treatment discontinuation. This, in turn, increases the risk of drug resistance and disease relapse [9].

To address these challenges, the WHO has emphasized the need for innovations in TB prevention and treatment, particularly through the development of novel therapeutic approaches [10,11]. Given the lengthy timelines and high costs associated with new drug development, optimizing formulations and delivery systems for existing anti-tuberculosis drugs presents a more practical strategy [12]. Drug delivery systems (DDSs), which encapsulate drugs within functional carriers to achieve targeted delivery, controlled release, and enhanced bioavailability, have emerged as a promising approach to improve treatment outcomes in TB [13,14].

Among various drug delivery carriers, polymeric micelles have garnered significant attention due to their unique structure and properties. Formed by the self-assembly of amphiphilic block copolymers, they offer advantages such as high drug-loading capacity, uniform particle size distribution (typically 10–200 nm), prolonged circulation in vivo, and strong passive targeting capabilities [15,16,17,18]. Currently, polymeric micelles as a drug delivery platform are progressively advancing toward clinical application, exemplified by FDA-approved cyclosporine A micellar eye drops (Cequa^®^) [19] and the paclitaxel micelles marketed by Shanghai YingZhong Pharma [20], providing a technical reference for their application in anti-tuberculosis therapy.

The drug of interest in this study, RPT, a semi-synthetic cyclopentyl antibiotic of rifamycin, demonstrates 2- to 10-fold greater antibacterial potency than rifampicin, along with an extended half-life and a more favorable safety profile [21,22]. Nevertheless, conventional oral RPT formulations are limited by extensive first-pass metabolism and variable bioavailability influenced by dietary intake, which hinder effective drug accumulation at osseous lesion sites [23]. Therefore, the development of non-invasive, advanced drug delivery systems that integrate both bone-targeting and sustained-release capabilities is of critical importance.

Approaches to bone targeting primarily fall into two categories: targeting bone cells or targeting the bone matrix [24]. Given that Mycobacterium tuberculosis can reside latently within macrophages rather than directly infect bone cells, this study focuses on hydroxyapatite (HA)—which constitutes 60–70% of the bone tissue by weight—as the target site [25]. As a bisphosphonate, ALN exhibits high-affinity chelation with calcium ions in HA through its bisphosphonate groups, thereby enabling active targeting toward the bone mineral phase. Furthermore, ALN possesses favorable aqueous solubility, and its primary amino group allows straightforward chemical modification, making it an ideal targeting ligand [26,27,28].

Building on this rationale, our research group developed a novel bone-targeted nanomicellar drug delivery system. This system is constructed using biocompatible and biodegradable poly(lactic-co-glycolic acid) (PLGA) as the hydrophobic core, which was further modified with a defined amount of methoxy-poly(ethylene glycol) (mPEG) to enhance hydrophilicity, resulting in a well-defined amphiphilic polymer capable of self-assembling into stable micelles. To confer active bone-targeting ability, ALN was conjugated to the terminal end of the PLGA block, ultimately yielding a functional carrier material [29]. In the present study, this nanomicelle system was employed to encapsulate RPT, and its physicochemical properties, drug release profile, and targeting capability were systematically evaluated. This design aims to achieve a synergistic integration of “bone targeting” and “sustained release,” thereby enhancing drug accumulation at the disease site, reducing systemic toxicity and dosing frequency, and potentially mitigating the risk of drug resistance. This strategy offers a new therapeutic approach for the treatment of bone tuberculosis and addresses the strategic need for innovative solutions in global tuberculosis control.

## 2. Materials and Methods

### 2.1. Materials

ALN-PLGA-mPEG was synthesized in our laboratory. Rifapentine (97% purity) and DIR (≥95.0% purity) were purchased from Shanghai Aladdin Biochemical Technology Co., Ltd. (Shanghai, China). Methanol (HPLC grade), potassium dihydrogen phosphate (≥99.5%), and acetonitrile (HPLC grade) were obtained from Sigma-Aldrich (St. Louis, MO, USA). Ultrafiltration centrifuge tubes (MWCO 3 kDa) were sourced from Millipore (Billerica, MA, USA). All other reagents were of analytical or chromatographic grade.

### 2.2. Animals

Male Institute of Cancer Research (ICR) mice (specific pathogen-free, 20–26 g) and male Sprague Dawley (SD) rats (specific pathogen-free, 200–300 g) were purchased from the Laboratory Animal Center of Xinjiang Medical University (Urumqi, Xinjiang, China).

### 2.3. Preparation and Characterization of Rifapentine-Loaded Bone-Targeting Micelles (ALN-PLGA-mPEG@RPT)

#### 2.3.1. Preparation of Drug-Loaded Micelles

Blank ALN-PLGA-mPEG micelles were first prepared following the protocol established by our research group [29]. The resulting polymer was dissolved in ultrapure water and sonicated using an ultrasonic processor (VCX500, SONICS, Newtown, CT, USA) under the following conditions: 30% amplitude, 3 s on/2 s off pulses, for 15 min, to obtain the blank micelle solution. Under continuous stirring with a magnetic stirrer (MS-H-S, Dragon Lab Instruments Co., Ltd., Beijing, China), an ethanolic solution of RPT was added dropwise, with the theoretical drug loading set at 10%, 20%, 30%, 40%, and 50% (*w*/*w*, relative to the polymer). Stirring was continued for 3 h. The mixture was then purified via ultrafiltration (MWCO 3000 Da). The retained solution (retentate) was collected and lyophilized using a freeze dryer (FDU-1200, Shanghai Ailang Instrument Co., Ltd., Shanghai, China) to obtain the drug-loaded ALN-PLGA-mPEG@RPT micelles. For comparison, non-targeted mPEG-PLGA@RPT micelles were prepared using the same procedure but without the ALN modification.

#### 2.3.2. Development and Validation of the Rifapentine Quantification Method

Chromatographic Conditions: The analysis was performed using an Agilent C18 column (4.6 × 250 mm, 5 μm, Agilent Technologies, Santa Clara, CA, USA) maintained at 25 ± 0.5 °C. Detection was carried out with a UV-Vis detector set at a wavelength of 254 nm. An isocratic elution mode was employed with a mobile phase consisting of methanol and 0.04 M phosphate buffer (70:30, *v*/*v*), delivered at a constant flow rate of 1.00 mL/min. The injection volume was 10.0 μL via an autosampler.

Establishment of the Standard Curve: A standard stock solution of RPT (100 µg/mL) was prepared in methanol. A series of standard working solutions with concentrations ranging from 5 to 50 µg/mL (5, 10, 20, 30, 40, 50 µg/mL) were obtained by serial dilution. These solutions were analyzed using a high-performance liquid chromatography (HPLC) system (UltiMate3000, Thermo Fisher Scientific, Waltham, MA, USA). The peak area for each concentration was recorded. The standard curve was constructed by plotting the peak area (A) against the corresponding concentration (C), and the linear regression equation was derived.

Method Validation: Methanol solutions of RPT at low, medium, and high concentrations (5, 20, and 40 μg/mL) were prepared to evaluate precision. Intra-day precision was assessed by analyzing replicates at 0, 3, 6, 9, and 12 h on the same day. Inter-day precision was evaluated by repeating the analysis over five consecutive days. Precision was expressed as the relative standard deviation (RSD) of the measured concentrations. To determine accuracy via spike recovery, low, medium, and high concentrations of RPT were spiked into blank ALN-PLGA-mPEG micelle solutions. The recovery rate was calculated using the formula: Recovery (%) = (Measured Concentration/Theoretical Concentration) × 100%, where the measured concentration was determined from the standard curve.

#### 2.3.3. Drug-Loading Performance and Physicochemical Characterization of the Micelles

The concentration of RPT in the ALN-PLGA-mPEG@RPT micelles was quantified using HPLC. The drug-loading capacity (DLC) was defined as the percentage of the mass of encapsulated RPT relative to the total mass of the drug-loaded micelles. The drug-loading efficiency (DLE), also referred to as encapsulation efficiency, represents the percentage of the actual amount of RPT encapsulated relative to the initial total amount of drug used in the preparation. During the formulation process, the filtrate obtained after ultrafiltration was collected. The concentration of free (unentrapped) drug in the filtrate was determined based on the RPT standard curve, which was then used to calculate the mass of unencapsulated drug.

DLC (%) = (Mass of encapsulated drug/Total mass of drug-loaded micelles) × 100%

DLE (%) = (Mass of encapsulated drug/Total initial drug input) × 100%

An appropriate amount of the optimized ALN-PLGA-mPEG@RPT micelles was dispersed in ultrapure water and sonicated (30% amplitude, 3 s on/2 s off pulses, 3 min) to form a micellar dispersion. The particle size and PDI were then determined using a nanoparticle size and zeta potential analyzer (Nano ZS90, Malvern Panalytical, Malvern, UK).

#### 2.3.4. Scanning Electron Microscopy (SEM) Analysis

The ALN-PLGA-mPEG@RPT sample was mounted on a conductive carbon tape and placed on the sample stage of an ion sputter coater (MC1000, Hitachi High-Tech, Tokyo, Japan). The sample was coated with a thin layer of gold for approximately 30 s. Subsequently, SEM imaging was performed using a SEM (SU8100, Hitachi High-Tech, Tokyo, Japan) operated at an accelerating voltage of 3.0 kV. The ultrastructural features of the sample were examined at a magnification of 200,000×.

#### 2.3.5. In Vitro Release Study

In vitro release characteristics of the drug-loaded micelles were investigated using dynamic dialysis. ALN-PLGA-mPEG@RPT, free RPT, and a physical mixture of ALN-PLGA-mPEG and RPT (containing an equivalent amount of RPT) were separately placed into dialysis bags (MWCO: 3.5 kDa). The bags were immersed in 100 mL of phosphate-buffered saline (PBS, pH 7.4) as the release medium and incubated in a full-color touch-screen dual-stack shaker (Model ZWYR-D2402, Shanghai Zhicheng Analytical Instrument Manufacturing Co., Ltd., Shanghai, China) under constant agitation (37 ± 0.5 °C, 100 rpm). The physical mixture control was included to confirm successful drug encapsulation and to evaluate the influence of micellar entrapment on the release behavior of RPT. Samples (1 mL) were withdrawn at predetermined time points (1, 2, 5, 8, 12, 24, 48, 72, and 120 h), and an equal volume of pre-warmed PBS was immediately replenished. All samples were filtered through a 0.22 μm microporous membrane prior to quantitative analysis of RPT content via HPLC. Each experiment was performed in triplicate, and data are presented as mean ± standard deviation. The cumulative release of RPT was calculated according to the following formula:

$$Cumulative\;Drug\;Release(\%)=\frac{Cn\times V+\sum_{1}^{n-1}Ci\times V^{\prime }}{M}\times 100$$

where C*n* is the drug concentration in the release medium at the *n*-th sampling point (μg/mL); V is the total volume of the release medium (mL); C*i* is the drug concentration in the release medium at the *i*-th sampling point (μg/mL); V′ is the volume of the release medium sample withdrawn each time (mL); M is the total amount of drug loaded in the dialysis bag (μg).

### 2.4. Evaluation of Bone-Targeting Capability

#### 2.4.1. Preparation of DIR Fluorescently Labeled Micelles

mPEG-PLGA was dissolved in ultrapure water and sonicated (30% amplitude, 3 s on/2 s off pulses, 3 min) to form micelles. To this solution, 2 mL of a prepared DIR solution (100 μmol/L) was added. The mixture was then incubated under constant shaking (200 rpm, 37 °C) in the dark for 3 h. After incubation, the mixture was purified by ultrafiltration. The retentate was collected to obtain DIR-labeled mPEG-PLGA micelles. Using the same procedure, DIR-labeled ALN-PLGA-mPEG micelles were also prepared.

#### 2.4.2. In Vivo Imaging Study

DIR-labeled mPEG-PLGA micelles and ALN-PLGA-mPEG micelles were first prepared and stored in the dark at low temperature for later use. Eighteen ICR mice were randomly divided into three groups (*n* = 6 per group): (1) the ALN-PLGA-mPEG fluorescent micelle group, (2) the mPEG-PLGA fluorescent micelle group, and (3) the free DIR group. Each group received an intravenous injection of the corresponding prepared micelle solution or free DIR solution via the tail vein. At pre-determined time points (before injection, and 1, 3, 6, 10, 24, 48, 72 h post-injection), the ICR mice were anesthetized and subjected to fluorescence imaging using a small animal in vivo imaging system (Lumina Series, PerkinElmer, Waltham, MA, USA). At 24 h post-injection, a subset of ICR mice was euthanized by cervical dislocation. The long bones of the limbs, vertebrae, and attached ribs were dissected. Residual soft tissue was carefully removed, and the bones were transferred to a 10 mL sterile culture dish for ex vivo fluorescence imaging. The imaging system parameters were set as follows: excitation wavelength, 745 nm; emission wavelength, 800 nm; cooling temperature, −90 °C; f-stop, 2; field of view, D-23 cm; objective lens height, 1.5 cm; exposure mode, auto.

#### 2.4.3. In Vivo Drug Distribution

Thirty-six male SD rats were randomly divided into two groups: (1) the free RPT group, and (2) the ALN-PLGA-mPEG@RPT micelle group. At predetermined time points (1, 24, and 48 h post-administration, *n* = 6 per time point per group), blood samples were collected from the orbital venous plexus. Subsequently, the rats were euthanized, and tissues including the liver and bone were harvested. The concentration of RPT in each sample was determined by HPLC. The targeting efficiency was assessed by calculating the tissue-to-plasma drug concentration ratio (C_tissue_/C_plasma_).

##### Sample Collection and Processing

Blood Samples: Approximately 0.4 mL of blood was collected from the orbital venous plexus of the SD rats. The blood samples were centrifuged at 3500 rpm for 5 min to obtain plasma. The plasma was then mixed with acetonitrile at a 1:2 (*v*/*v*) ratio, vortexed at 2000 rpm for 2 min, and centrifuged again (4 °C, 13,000 rpm, 10 min). The resulting supernatant was filtered through a 0.22 μm organic membrane filter and then subjected to HPLC analysis.

Tissue Samples: After blood collection, the SD rats were euthanized, and the liver and femur were harvested. The liver tissue was rinsed with pre-cooled saline to remove residual blood and weighed. It was then minced into approximately 1 mm^3^ pieces. The tissue fragments were homogenized in saline (1:5, *w*/*v*) using a variable high-speed homogenizer (FSH-2A, Jinyi Instrument Technology Co., Ltd., Changzhou, Jiangsu, China) with two 30 s cycles and intermittent cooling. Subsequently, the homogenate was further disrupted using an ultrasonic cell disruptor (JY92-2D, Scientz Biotechnology Co., Ltd., Ningbo, Zhejiang, China) under the following conditions: 30% amplitude, 3 s on/2 s off pulses, for 3 min. Bone tissue was frozen with liquid nitrogen and pulverized. The liver or bone tissue homogenate was mixed with acetonitrile, vortexed at 2000 rpm for 2 min, and centrifuged (4 °C, 13,000 rpm, 10 min). The supernatant was collected, filtered through a 0.22 μm organic membrane filter, and analyzed by HPLC.

##### Development and Validation of the RPT Quantification Method in Biological Samples

Chromatographic Conditions: As described in Section 2.3.2 above.

Establishment of the Standard Curve: A standard stock solution of RPT was serially diluted with blank rat plasma to obtain a series of standard solutions at concentrations of 0.25, 0.5, 1, 5, 15, and 25 µg/mL. These samples were processed according to the method described in Section Sample Collection and Processing and then analyzed by HPLC. A standard curve was constructed by plotting the peak area (A) against the corresponding concentration (C), and the linear regression equation was derived.

Method Validation: Plasma samples spiked with RPT at low, medium, and high concentrations (0.4, 10, and 20 μg/mL) were prepared. Intra-day precision was assessed by analyzing five replicates of each concentration level at 0, 3, 6, 9, and 12 h on the same day. Inter-day precision was evaluated by repeating the analysis over five consecutive days. Precision was expressed as the RSD of the measured concentrations. Accuracy was determined using a standard addition (spike-recovery) method. The recovery rate was calculated as the percentage of the measured concentration (calculated from the standard curve) relative to the theoretical spiked concentration.

### 2.5. Histopathological Analysis

To evaluate the in vivo biosafety of the ALN-PLGA-mPEG@RPT nanomicelles, twelve male SD rats were randomly assigned to two groups (*n* = 6 per group): (1) the ALN-PLGA-mPEG@RPT micelle group, and (2) the saline control group. At 48 h after intravenous administration via the tail vein, the rats were deeply anesthetized with 2% pentobarbital sodium. Perfusion fixation was performed via cannulation of the aorta through the left ventricle, with the right atrium opened. Rapid perfusion with 150 mL of physiological saline was followed by perfusion with 300 mL of tissue fixative (4% paraformaldehyde). The heart, kidneys, spleen, lungs, and liver were carefully excised and post-fixed in the same fixative for 72 h. The fixed tissue blocks were then dehydrated through a graded ethanol series, cleared in xylene, infiltrated with paraffin, and embedded. The paraffin blocks were sectioned serially to a thickness of 3–5 μm. Following deparaffinization and rehydration, the sections were stained with hematoxylin and eosin (H&E). The histological morphology was examined and images were captured using an optical microscope (ECLIPSE E100, Nikon, Tokyo, Japan).

### 2.6. Statistical Analysis

The data are presented as the mean ± standard deviation. Graphs were plotted using GraphPad Prism software (version 8.0). Differences between two groups were analyzed using Student’s *t*-test, while comparisons among multiple groups were performed using one-way analysis of variance (one-way ANOVA). All statistical analyses were conducted with SPSS software (version 29.0). A *p*-value of less than 0.05 was considered statistically significant.

## 3. Results and Discussion

### 3.1. Preparation and Characterization of ALN-PLGA-mPEG@RPT

#### 3.1.1. Development and Validation of the RPT Quantification Method

A standard calibration curve was established by linear regression analysis with the peak area (A) as the dependent variable and the concentration (C, µg/mL) as the independent variable. The resulting regression equation was A = (281.75 ± 0.68) C − (125.37 ± 23.12) (*n* = 3), with a correlation coefficient (R^2^) of 0.9995. This RPT quantification method exhibited excellent linearity over the concentration range of 5–50 μg/mL. An R^2^ value approaching 1 indicates high sensitivity and reliability of the assay.

In precision studies, both intra-day and inter-day relative standard deviations (RSDs) for RPT at low, medium, and high concentrations (5, 20, and 40 µg/mL) were below 2% (Table 1), demonstrating excellent repeatability and minimal variability over time and across analytical runs, thereby minimizing random error in quantification. The average recovery rates for the three concentrations fell within the acceptable range of 95–105%, with RSDs well below 2% (Table 2), confirming that the quantitative results are in high agreement with the true values and validating the accuracy and reliability of the method.

In summary, this analytical method exhibits satisfactory linearity, precision, and accuracy, meeting the stringent requirements for methodological validation in pharmaceutical analysis. It thus provides a robust methodological foundation for subsequent quality control of the formulation and the determination of RPT content.

#### 3.1.2. Drug-Loading Performance, Feeding Ratio Optimization, and Physicochemical Characterization of the Micelles

This study investigated the effect of different drug-to-polymer feeding ratios on the RPT loading performance of ALN-PLGA-mPEG nanomicelles. As presented in Table 3, with an increasing feeding ratio of RPT, the DLC showed an upward trend, while the DLE gradually decreased. When the feeding ratio (polymer: drug, *w*/*w*) reached 10:4, the DLC peaked at (16.74 ± 0.51)%, but the DLE dropped to (50.27 ± 1.91)%. This phenomenon indicates the existence of a saturation point for the drug-loading capacity of the micellar material. At lower drug-feeding amounts, the PLGA hydrophobic core of the micelles provided ample space to accommodate drug molecules, resulting in a higher DLE. When the drug feed was continuously increased (e.g., to 10:4 and 10:5 ratios), the DLC plateaued while the DLE decreased significantly, suggesting that the drug-loading capacity of the micelles had reached its upper limit. Excess drug could not be effectively encapsulated and remained in a free form. Consequently, a feeding ratio of 10:4 was selected as optimal, achieving a high DLC while maintaining an acceptable DLE. Future studies could aim to further enhance the encapsulation efficiency by optimizing preparation parameters such as sonication time, stirring rate, or the introduction of co-solvents.

The optimized ALN-PLGA-mPEG@RPT nanomicelles exhibited an average particle size of 101.90 ± 4.17 nm and a PDI of 0.242 ± 0.021. The PDI, a key indicator for assessing the size uniformity of a nano-delivery system, ranges from 0 to 1. A PDI value below 0.3 indicates a system with excellent monodispersity [30,31].

#### 3.1.3. SEM Analysis

**Figure 1:**
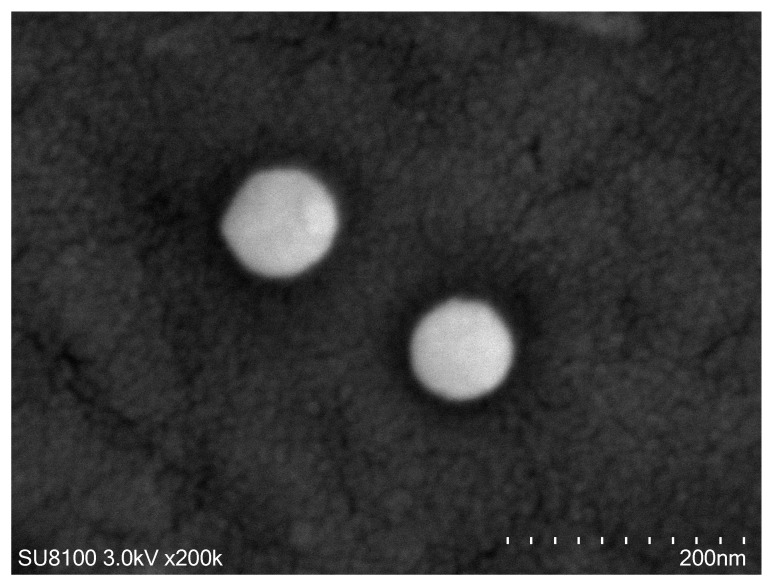
SEM image of ALN-PLGA-mPEG@RPT (×200,000).

As shown in Figure 1, the prepared ALN-PLGA-mPEG@RPT nanomicelles exhibited a predominantly spherical or near-spherical morphology with a uniform particle size distribution around 100 nm. The micellar surfaces appeared smooth, without noticeable cracks or aggregation, demonstrating favorable sphericity and stability during the preparation process. Furthermore, no discernible drug crystals were observed in the images, preliminarily suggesting that RPT is dispersed within the micellar core in an amorphous or molecularly dissolved state. This characteristic is conducive to achieving sustained drug release and enhancing bioavailability [32,33].

#### 3.1.4. In Vitro Release Profile

**Figure 2:**
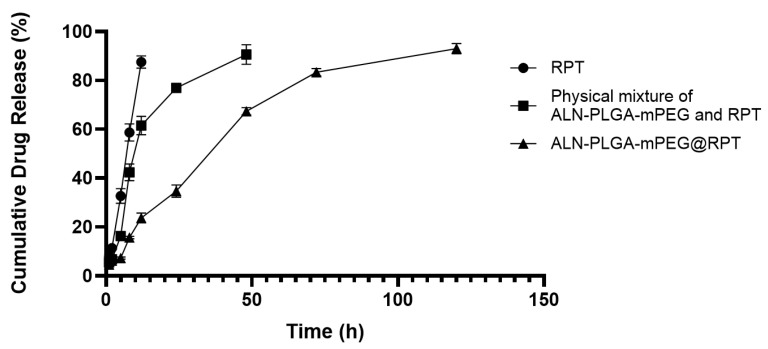
In vitro cumulative release profile of RPT (*n* = 3).

Figure 2 visually demonstrates the differences in release rates among the three formulations. To further elucidate the release characteristics and underlying mechanisms, the in vitro release data were systematically analyzed by fitting to several kinetic models: zero-order, first-order, Higuchi, and Korsmeyer–Peppas models [34,35].

Free RPT exhibited a typical rapid release profile, with a cumulative release of 87.92 ± 2.22% within 12 h. Model fitting identified the zero-order model (R^2^ = 0.99, Qt = 7.32t + 0.37) as the best fit, indicating a near-constant release rate. This behavior originates from a dissolution–diffusion dominated mechanism, where drug particles dissolve quickly and diffuse into the release medium without the restraining effect of a polymeric matrix.

The release rate of the physical mixture (ALN-PLGA-mPEG + RPT) was intermediate between that of free drug and the micellar formulation, reaching a cumulative release of 90.82 ± 3.41% at 48 h. Both the Higuchi model (R^2^ = 0.99, Qt = 13.10√t − 7.60) and the Korsmeyer–Peppas model (R^2^ = 0.99, n ≈ 0.83) provided excellent fits, suggesting a non-Fickian diffusion mechanism dominated by drug diffusion, with potential minor influence from polymer erosion.

The micellar formulation (ALN-PLGA-mPEG@RPT) displayed pronounced sustained-release characteristics, with a cumulative release of 92.98 ± 1.47% over 120 h and only 24.50 ± 2.11% released at 12 h. The Higuchi model was the best fit (R^2^ = 0.99, Qt = 8.50√t − 3.80), and the Korsmeyer–Peppas model yielded an exponent n ≈ 0.75, consistent with a non-Fickian diffusion-dominated release mechanism. The core–shell structure of the micelles imposes spatial hindrance, restricting rapid drug diffusion and thereby enabling a slow and gradual release profile [33,35].

The intermediate release rate observed for the physical mixture, positioned between the free RPT and the micellar formulation, helps confirm that the sustained-release behavior of ALN-PLGA-mPEG@RPT nanomicelles originates primarily from micelle formation and the entrapment of drugs within the core–shell structure, rather than from nonspecific interactions or the mere presence of the polymer. This finding provides a formulation basis for achieving local retention and prolonged action in the treatment of bone tuberculosis.

Although bone infection sites often present an acidic microenvironment [36], the current release study was conducted solely at pH 7.4. This is because the standard PLGA-mPEG conjugate, linked via ester bonds, lacks intrinsic pH responsiveness. Achieving pH-dependent release would require specific chemical modifications, such as the incorporation of hydrazone bonds. The degradation of the current system relies primarily on the hydrolysis of ester bonds, a process that may be marginally influenced by increased acidity but does not constitute its main release mechanism [37,38]. The potential influence of pH on the release profile of this micelle system warrants further investigation in subsequent studies.

### 3.2. Bone-Targeting Capability

#### 3.2.1. In Vivo Imaging Analysis of Bone-Targeting Performance

**Figure 3:**
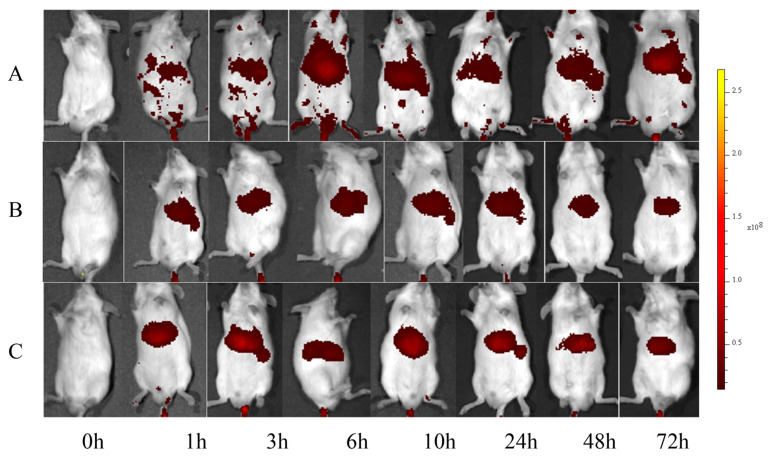
Fluorescence scan results of fluorescent nanomicelles using a small animal in vivo imaging system. (**A**) ALN-PLGA-mPEG fluorescent micelle group; (**B**) mPEG-PLGA fluorescent micelle group; (**C**) Free DIR group.

**Figure 4:**
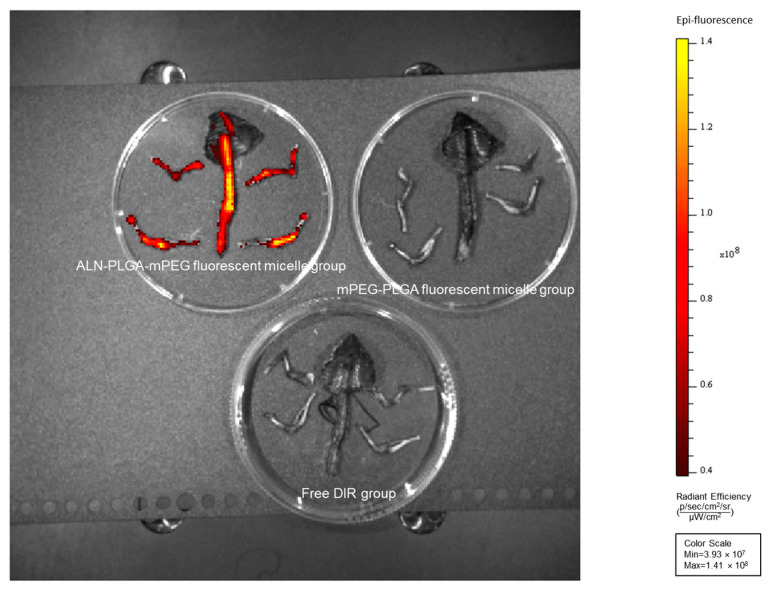
Ex vivo bone tissue fluorescence imaging scan of fluorescent nanomicelles.

The in vivo distribution of DIR-labeled nanoparticles was successfully tracked using a small animal in vivo imaging system. The results showed that after intravenous injection, the fluorescence signals in the mPEG-PLGA fluorescent micelle group and the free DIR group were predominantly concentrated in the liver. In contrast, the ALN-PLGA-mPEG fluorescent micelle group exhibited significant fluorescence signals in both the limbs and liver (Figure 3). Furthermore, ex vivo fluorescence scanning of isolated bone tissues (limb bones, spine, and rib cage) revealed that the bone tissue fluorescence intensity in the ALN-PLGA-mPEG micelle group was significantly higher than that in both the mPEG-PLGA micelle group and the free DIR group (Figure 4). These results confirm that the introduction of the ALN ligand significantly enhances the active bone-targeting capability of the nanocarrier.

#### 3.2.2. Quantitative Assay Validation for RPT in Biological Samples

**Figure 5:**
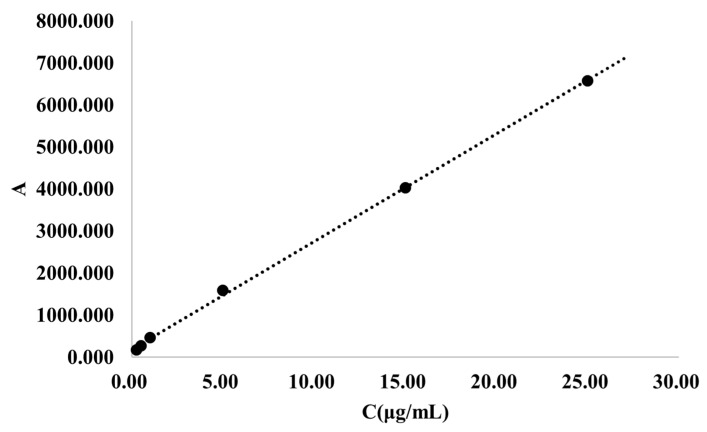
Standard curve of RPT in biological samples determined by HPLC (*n* = 3).

To accurately determine the concentration of RPT in rat biological samples, a quantitative analysis method based on HPLC was established. The standard curve demonstrated good linearity within the range of 0.25 to 25 μg/mL, with a regression equation of A = 256.29C + 187.59 (R^2^ = 0.9993) (Figure 5). Method validation results indicated that the intra-day precision (RSD: 2.85–6.22%), inter-day precision (RSD: 2.35–9.42%), and extraction recovery (99.41–101.37%) all met the quality control criteria for bioanalytical methods (Table 4, Table 5 and Table 6). This reliable analytical method provided a technical foundation for the subsequent drug distribution study.

#### 3.2.3. In Vivo Distribution Profile of RPT in SD Rats

The tissue distribution of RPT in SD rats was determined by HPLC analysis at 1, 24, and 48 h post-administration (Table 7). In the group administered free RPT, the drug was predominantly distributed to the liver at 1 h, with a liver-to-blood ratio of 2.94 ± 0.43. In contrast, the concentration in bone tissue was minimal, being only 0.23 ± 0.05-fold of the plasma concentration. This distribution pattern, characterized by liver > blood > bone, persisted at both 24 and 48 h, with bone drug levels remaining consistently lower than those in the liver and blood throughout the study period.

Conversely, a markedly different profile was observed for the group treated with the ALN-PLGA-mPEG@RPT nanomicelles. At 1 h post-injection, the drug concentration in bone tissue had already approached the plasma level, yielding a bone-to-blood ratio of 0.95 ± 0.18. Simultaneously, the RPT concentration in the liver was significantly lower than that in the free RPT group (*p* < 0.05). By 24 and 48 h, the distribution hierarchy for the targeted group had shifted to liver > bone > blood. Notably, at the 48 h time point, the bone drug concentration reached 1.93 ± 0.30-fold of the plasma concentration, whereas it was merely 0.33 ± 0.07-fold in the non-targeted group. Quantitative analysis of these data unequivocally demonstrates that the ALN-PLGA-mPEG nanomicelle system profoundly altered the in vivo distribution pattern of RPT, leading to a substantial enhancement of drug accumulation in bone tissue.

The dramatic shift in biodistribution can be attributed to the synergistic design of the ALN-PLGA-mPEG nanomicellar system. First, the ‘stealth’ effect and prolonged circulation conferred by mPEG: The hydrophilic poly(ethylene glycol) (mPEG) corona enhances the hydrophilicity of the PLGA core and provides steric stabilization, effectively reducing opsonization and subsequent clearance by the mononuclear phagocyte system. This extends the systemic circulation time of the nanomicelles, providing a critical window for bone targeting [39,40,41]. Second, the active bone-targeting function of ALN: The alendronate (ALN) moieties conjugated to the micelle surface exhibit high affinity for hydroxyapatite, the major mineral component of bone, enabling active targeting and retention at the osseous site [26,27,28]. Finally, a synergistic effect is achieved: The combination of prolonged circulation (enabling more micelles to reach bone vasculature) and active targeting (promoting specific binding and uptake) facilitates the effective traversal of the blood-bone marrow barrier and culminates in the selective enrichment and prolonged retention of the drug within bone tissue.

In conclusion, the data from this study provide robust evidence that the ALN-PLGA-mPEG nanomicelle system successfully redirects RPT to skeletal tissues, aligning perfectly with the strategic objectives of bone-targeted nanomedicine. To further elucidate the dynamic in vivo distribution and elimination characteristics of this formulation and to advance its translational potential, conducting a systematic pharmacokinetic study with dense multi-timepoint sampling will be the essential next step.

### 3.3. Histopathological

**Figure 6:**
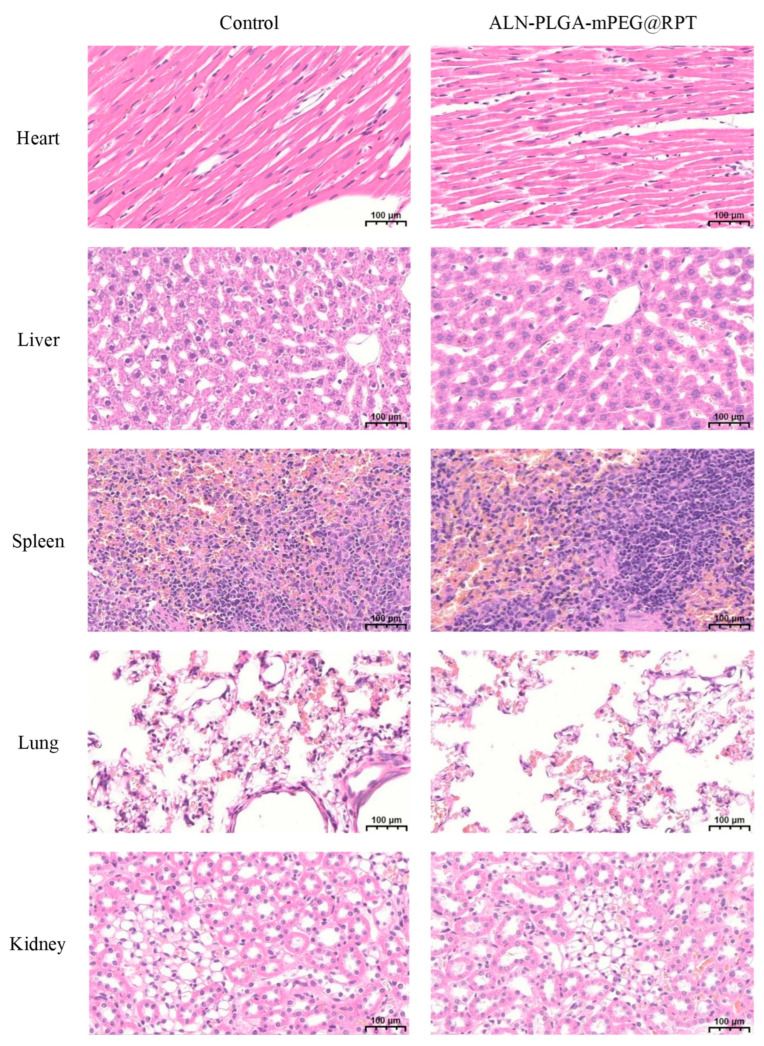
Representative H&E-stained images of major rat organs from the ALN-PLGA-mPEG@RTP nanomicelle-treated group and the saline control group (magnification, 20×; *n* = 6).

Histopathological evaluation of major organs (heart, spleen, lung, kidney, and liver) from SD rats was performed via H&E staining at 48 h post-administration. Compared with the saline control group, the ALN-PLGA-mPEG@RPT nanomicelle-treated group showed no discernible morphological alterations, necrosis, or signs of inflammation in any of the examined organs (Figure 6). These findings indicate favorable biocompatibility of ALN-PLGA-mPEG@RPT at the administered dose. Furthermore, PLGA—a biodegradable polymer approved by both the FDA and EMA for use in medical formulations—possesses a well-established safety profile. Its degradation products, lactic acid and glycolic acid, are metabolically eliminated from the body [42,43,44]. Nevertheless, further long-term toxicity studies remain warranted to comprehensively assess its safety.

## 4. Conclusions

In this study, we successfully developed and systematically evaluated an ALN-modified bone-targeted nanomicelle system for the targeted delivery of RPT, aiming to address the challenges of low drug accumulation and systemic toxicity in the treatment of bone tuberculosis. The formulation-optimized ALN-PLGA-mPEG@RPT nanomicelles exhibited favorable particle size, zeta potential, DLC, and DLE. More importantly, they demonstrated sustained release characteristics in vitro, with only approximately 25% of RPT released within 12 h—a marked contrast to the rapid release of free RPT—suggesting the potential for reduced dosing frequency. The significant difference in release compared to the physical mixture further corroborated the successful formation of ALN-PLGA-mPEG@RPT micelles and the encapsulation of the drug within their core–shell structure. In vivo imaging and biodistribution studies in this work demonstrated significant accumulation of the targeted micelles in bone tissue, along with reduced deposition in non-target organs such as the liver. These findings are consistent with and further corroborate our previous in vitro HA binding assays using blank micelles, which confirmed a substantially enhanced bone affinity conferred by ALN modification. Furthermore, histopathological analysis revealed no significant toxic damage in the major organs, indicating the favorable biocompatibility of this delivery system.

The combined strategy of “long-acting drug + sustained release + active bone targeting” embodied by the ALN-PLGA-mPEG@RPT micelles can significantly enhance drug delivery to bone tissue, optimize dosing regimens, and improve patient compliance, thereby offering a highly promising strategy for the precise treatment of bone-related diseases such as bone tuberculosis. Future work will focus on evaluating its long-term efficacy and safety in disease models to facilitate the translational development of this platform toward clinical application.

## 5. Patents

Li, S. S.; Wang, W. L.; Han, Y. L.; Li, X. X.; Song, X. H. Bone-Targeting Nanomicelles Loaded with Rifapentine, Their Preparation Method and Application. Xinjiang Uygur Autonomous Region, China: CN120983359A, 21 November 2025.

## Figures and Tables

**Table 1:** Results of the precision evaluation for RPT by HPLC (*n* = 5).

C (μg/mL)	Intra-Day RSD (%)	Inter-Day RSD (%)
5	0.62	1.92
20	0.58	1.28
40	0.39	1.09

**Table 2:** Results of the recovery rate evaluation for RPT by HPLC (*n* = 3).

C (μg/mL)	Average Recovery (%)	RSD (%)
5	100.39	1.11
20	101.50	0.97
40	100.42	0.65

**Table 3:** Determination results of DLE and DLC for RPT (mean ± SD, *n* = 3).

Feeding Ratio (Micelle:RPT, mg:mg)	DLC (%)	DLE (%)
10:1	8.01 ± 0.33	87.08 ± 1.94
10:2	12.75 ± 0.62	73.06 ± 2.11
10:3	13.54 ± 0.56	52.22 ± 1.72
10:4	16.74 ± 0.51	50.27 ± 1.91
10:5	16.69 ± 0.66	40.07 ± 1.63

**Table 4:** Intra-day precision results of RPT in biological samples determined by HPLC.

Time (h)	Low Concentration	Medium Concentration	High Concentration
0	0.39	10.2	19.6
3	0.41	10.4	20.4
6	0.38	9.5	20.0
9	0.43	9.2	20.7
12	0.44	9.8	19.3
Mean	0.41	9.82	20
RSD (%)	6.22	5.01	2.85

**Table 5:** Inter-day precision results of RPT in biological samples determined by HPLC.

Time (d)	Low Concentration	Medium Concentration	High Concentration
1	0.43	9.4	20.1
2	0.38	9.6	20.4
3	0.38	9.8	19.9
4	0.37	9.2	19.4
5	0.33	9.7	19.3
Mean	0.38	9.54	19.82
RSD (%)	9.42	2.53	2.35

**Table 6:** Extraction recovery results of RPT in biological samples determined by HPLC.

	Low Concentration	Medium Concentration	High Concentration
Recovery (%)	101.37 ± 7.66	99.41 ± 2.97	100.56 ± 2.74

**Table 7:** Distribution ratios of RPT in various SD rat tissues (*n* = 6, mean ± SD).

	RPT			ALN-PLGA-mPEG@RPT		
			${\bar{C}}_{\mathrm{Tissue}}$:${\bar{C}}_{\mathrm{Plasma}}$			
	Sampling Time (h)			Sampling Time (h)		
	1	24	48	1	24	48
Blood	1.00	1.00	1.00	1.00	1.00	1.00
Liver	2.94 ± 0.43	2.66 ± 0.31	2.75 ± 0.47	2.19 ± 0.22	2.05 ± 0.23	1.96 ± 0.37
Bone	0.23 ± 0.05	0.37 ± 0.07	0.33 ± 0.07	0.95 ± 0.18	1.56 ± 0.26	1.93 ± 0.30

## Data Availability

The original contributions presented in this study are included in the article. Further inquiries can be directed to the corresponding authors.

## Abbreviations

The following abbreviations are used in this manuscript:

ALN	Alendronate Sodium
DDS	Drug Delivery System
DIR	1,1′-Dioctadecyl-3,3,3′,3′-tetramethylindotricarbocyanine Iodide
DLC	Drug-Loading Capacity
DLE	Drug-Loading Efficiency
HA	Hydroxyapatite
H&E	Hematoxylin and Eosin
HPLC	High Performance Liquid Chromatography
mPEG	Methoxy Polyethylene Glycol
MWCO	Molecular Weight Cut-Off
PBS	Phosphate-Buffer Saline
PDI	Polydispersity Index
PLGA	Polylactic-co-glycolic Acid
rpm	Revolutions Per Minute
RPT	Rifapentine
TB	Tuberculosis
